# Poly[μ_2_-aqua-aqua-μ_4_-pyridine-2,4-dicarboxyl­ato-strontium]

**DOI:** 10.1107/S160053680902683X

**Published:** 2009-07-15

**Authors:** Janet Soleimannejad, Yaghoub Mohammadzadeh, Hossein Aghabozorg, Zohreh Derikvand

**Affiliations:** a Department of Chemistry, Faculty of Science, Ilam University, Ilam, Iran; b Faculty of Chemistry, Islamic Azad University, North Tehran Branch, Tehran, Iran; c Department of Chemistry, Faculty of Science, Islamic Azad University, Khorramabad Branch, Khorramabad, Iran

## Abstract

In the title polymeric complex, [Sr(C_7_H_3_NO_4_)(H_2_O)_2_]_*n*_, the Sr^II^ atom is eight-coordinated by four O atoms and one N atom of four pyridine-2,4-dicarboxyl­ate (py-2,4-dc) ligands and three O atoms of three coordinated water mol­ecules in a dodeca­hedral geometry. These units are connected *via* the carboxyl­ate O atoms and water mol­ecules, building polymeric layers parallel to (100). In the crystal structure, non-covalent inter­actions consisting of O—H⋯O hydrogen bonds and π–π stacking inter­actions [centroid–centroid distances = 3.862 (17) and 3.749 (17) Å] connect the various components, forming a three-dimensional structure.

## Related literature

For related structures, see: Aghabozorg, Manteghi & Sheshmani (2008 ▶); Aghabozorg, Nemati *et al.* (2008 ▶); Liang (2008 ▶); Soleimannejad *et al.* (2007 ▶).
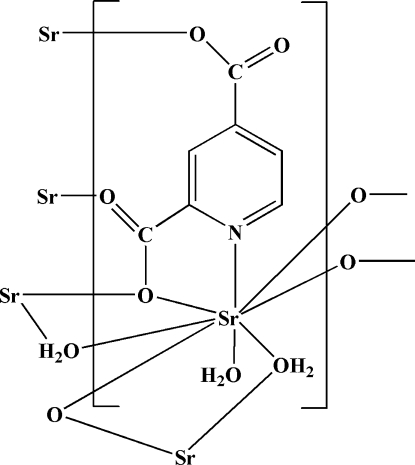


## Experimental

### 

#### Crystal data

[Sr(C_7_H_3_NO_4_)(H_2_O)_2_]
*M* *_r_* = 288.76
Monoclinic, 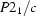

*a* = 6.8860 (5) Å
*b* = 19.7801 (13) Å
*c* = 6.5642 (4) Å
β = 91.892 (5)°
*V* = 893.59 (10) Å^3^
*Z* = 4
Mo *K*α radiation
μ = 6.04 mm^−1^
*T* = 296 K
0.08 × 0.05 × 0.05 mm

#### Data collection

Bruker SMART 1000 diffractometer
Absorption correction: multi-scan (*SADABS*; Sheldrick, 1996 ▶) *T*_min_ = 0.560, *T*_max_ = 0.752
6370 measured reflections
2321 independent reflections
1795 reflections with *I* > 2σ(*I*)
*R*_int_ = 0.042

#### Refinement

*R*[*F* ^2^ > 2σ(*F* ^2^)] = 0.030
*wR*(*F* ^2^) = 0.070
*S* = 1.03
2321 reflections
136 parameters
H-atom parameters constrained
Δρ_max_ = 0.76 e Å^−3^
Δρ_min_ = −0.59 e Å^−3^

### 

Data collection: *SMART* (Bruker, 1998 ▶); cell refinement: *SAINT* (Bruker, 1998 ▶); data reduction: *SAINT*; program(s) used to solve structure: *SHELXS97* (Sheldrick, 2008 ▶); program(s) used to refine structure: *SHELXL97* (Sheldrick, 2008 ▶); molecular graphics: *SHELXTL* (Sheldrick, 2008 ▶); software used to prepare material for publication: *SHELXTL*.

## Supplementary Material

Crystal structure: contains datablocks I, global. DOI: 10.1107/S160053680902683X/pv2167sup1.cif

Structure factors: contains datablocks I. DOI: 10.1107/S160053680902683X/pv2167Isup2.hkl

Additional supplementary materials:  crystallographic information; 3D view; checkCIF report

## Figures and Tables

**Table 1:** Hydrogen-bond geometry (Å, °)

*D*—H⋯*A*	*D*—H	H⋯*A*	*D*⋯*A*	*D*—H⋯*A*
O5—H5*B*⋯O4^i^	0.85	1.95	2.759 (3)	158
O5—H5*A*⋯O4^ii^	0.85	1.92	2.730 (3)	160
O5—H5*A*⋯O3^ii^	0.85	2.37	3.051 (3)	137
O6—H6*B*⋯O3^iii^	0.85	2.12	2.958 (3)	169
O6—H6*A*⋯O4^iv^	0.85	2.10	2.833 (3)	144

Symmetry codes: (i) 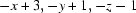; (ii) 
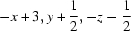
; (iii) 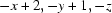; (iv) 
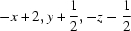
.

**Fig. 1.**
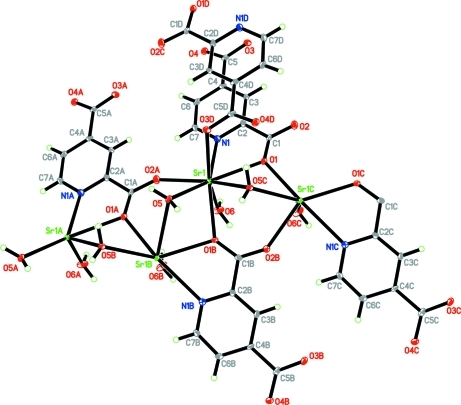
The molecular structure of polymeric complex, [Sr(C7H3NO4)(H2O)2]n. Displacement ellipsoids are drawn at the 50% probability level. Symmetry codes: (A) x, y, z - 1; (B) x, -y + 3/2, z - 1/2; (C) x, -y + 3/2, z + 1/2; (D) -x + 3, -y + 1, -z.

**Fig. 2.**
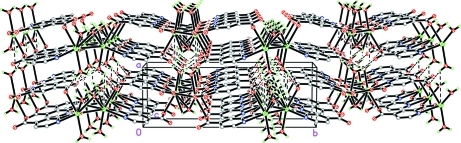
Crystal packing of the title complex, dashed lines indicate hydrogen bonds.

## supplementary crystallographic information

### Comment

We have previously reported two complexes of Sr^II^ with pyridine-3,5-dicarpoxylic and pyridine-2,6-dicarboxylic acid [Sr(C_7_H_3_NO_4_)(H_2_O)_4_]_n_ (Aghabozorg *et al.*, 2008; Aghabozorg, Manteghi *et al.*, 2008) and (C_10_H_10_N_2_)[Sr(C_7_H_3_NO_4_)_2_(H_2_O)_3_].3H_2_O (Soleimannejad *et al*, 2007). The co-crystal of this acid has been published C_7_H_5_NO_4_.C_3_H_7_NO_3_ (Liang, 2008).

Here we repot on the crystal structure of the title polymeric complex which is a two-dimensional polymer (Fig. 1). The Sr–O distances are in the range of 2.511 (2)–2.688 (2) Å, and the bond angles and bond distances around Sr^II^ atom show that the coordination environment of Sr^II^ atom is distored dodecahedron.

The carboxylate groups from py-2,4-dc (where py = pyridine and dc = dicarboxylate) link four Sr^II^ centers by four O atoms (O1^i^, O1^ii^, O2 and O3), [symmetry cods: (i) *x*, *y*, 1 + *z*, (ii), *x*,1.5 - *y*, 1/2 + *z*.] and one N1 atom result in the formation of two-dimensional polymeric chain in the crystal structure. There are a number of O–H···O hydrogen bonds with distances ranging 2.759 (3) Å to 3.052 (3) Å (Table 1). In the crystal structure there are many pores that can be used for storage of gas and elimination of guest molecules. Noncovalent interactions consist of hydrogen bonding and π–π stacking interactions with centroied-centroied distances [3.862 (17) Å and 3.749 (17) Å] connect the various components to form a supramolecular structure (Fig. 2).

### Experimental

An aqueous solution of 4,4'-bipyridine (100 mg, 2 mmol) and pyridine-2,4-dicarboxylic acid (53 mg, 1 mmol) was refluxed for an hour. A solution of Sr(NO_3_)_2 _(134 mg, 0.5 mmol) in water (3 ml) was added to the solution and refluxed for an hour. Colorless crystals were obtained after one week by the slow evaporation of the solvent at room temperature.

### Refinement

The H atoms of the water molecule these were located from low theta Fourier maps and all H-atoms were included in calculated positions and refined by a constrained rigid type geometry in a riding mode with O—H = 0.85 Å and C—H = 0.93 Å and *U*_iso_(H) = 1.2*U*_eq_(parent O or C-atom).

### Crystal data

**Table:**

[Sr(C_7_H_3_NO_4_)(H_2_O)_2_]	*F*(000) = 568
*M**_r_* = 288.76	*D*_x_ = 2.146 Mg m^−^^3^
Monoclinic, *P*2_1_/*c*	Mo *K*α radiation, λ = 0.71073 Å
Hall symbol: -P 2ybc	Cell parameters from 1756 reflections
*a* = 6.8860 (5) Å	θ = 4.4–28.4°
*b* = 19.7801 (13) Å	µ = 6.04 mm^−^^1^
*c* = 6.5642 (4) Å	*T* = 296 K
β = 91.892 (5)°	Plate, colourless
*V* = 893.59 (10) Å^3^	0.08 × 0.05 × 0.05 mm
*Z* = 4	

### Data collection

**Table:**

Bruker SMART 1000 diffractometer	2321 independent reflections
Radiation source: fine-focus sealed tube	1795 reflections with *I* > 2σ(*I*)
graphite	*R*_int_ = 0.042
Detector resolution: 100 pixels mm^-1^	θ_max_ = 28.9°, θ_min_ = 4.1°
ω scans	*h* = −9→9
Absorption correction: multi-scan (*SADABS*; Sheldrick, 1996)	*k* = −22→26
*T*_min_ = 0.560, *T*_max_ = 0.752	*l* = −8→8
6370 measured reflections	

### Refinement

**Table:**

Refinement on *F*^2^	Primary atom site location: structure-invariant direct methods
Least-squares matrix: full	Secondary atom site location: difference Fourier map
*R*[*F*^2^ > 2σ(*F*^2^)] = 0.030	Hydrogen site location: inferred from neighbouring sites
*wR*(*F*^2^) = 0.070	H-atom parameters constrained
*S* = 1.03	*w* = 1/[σ^2^(*F*_o_^2^) + (0.0326*P*)^2^ + 0.0723*P*] where *P* = (*F*_o_^2^ + 2*F*_c_^2^)/3
2321 reflections	(Δ/σ)_max_ = 0.001
136 parameters	Δρ_max_ = 0.76 e Å^−^^3^
0 restraints	Δρ_min_ = −0.58 e Å^−^^3^

### Special details

**Table:**

Geometry. All esds (except the esd in the dihedral angle between two l.s. planes) are estimated using the full covariance matrix. The cell esds are taken into account individually in the estimation of esds in distances, angles and torsion angles; correlations between esds in cell parameters are only used when they are defined by crystal symmetry. An approximate (isotropic) treatment of cell esds is used for estimating esds involving l.s. planes.
Refinement. Refinement of F^2^ against ALL reflections. The weighted R-factor wR and goodness of fit S are based on F^2^, conventional R-factors R are based on F, with F set to zero for negative F^2^. The threshold expression of F^2^ > 2sigma(F^2^) is used only for calculating R-factors(gt) etc. and is not relevant to the choice of reflections for refinement. R-factors based on F^2^ are statistically about twice as large as those based on F, and R- factors based on ALL data will be even larger.

### Fractional atomic coordinates and isotropic or equivalent isotropic displacement parameters (Å^2^)

**Table:**

	*x*	*y*	*z*	*U*_iso_*/*U*_eq_	
Sr1	1.41849 (4)	0.689568 (14)	−0.36705 (4)	0.00741 (8)	
N1	1.2631 (4)	0.57709 (13)	−0.2058 (4)	0.0096 (5)	
O1	1.3664 (3)	0.68543 (10)	0.0213 (3)	0.0094 (4)	
O2	1.3348 (3)	0.62646 (11)	0.3099 (3)	0.0127 (5)	
O3	1.2609 (3)	0.37523 (11)	0.2554 (3)	0.0112 (5)	
O4	1.1524 (3)	0.33289 (11)	−0.0422 (3)	0.0103 (4)	
O5	1.6546 (3)	0.72846 (10)	−0.6459 (3)	0.0103 (4)	
H5B	1.7139	0.7007	−0.7206	0.012*	
H5A	1.7156	0.7648	−0.6172	0.012*	
O6	1.0607 (3)	0.71224 (12)	−0.3855 (4)	0.0172 (5)	
H6B	0.9789	0.6826	−0.3505	0.021*	
H6A	1.0167	0.7507	−0.3525	0.021*	
C1	1.3325 (4)	0.63210 (15)	0.1201 (5)	0.0088 (6)	
C2	1.2865 (4)	0.56895 (15)	−0.0027 (4)	0.0085 (6)	
C3	1.2705 (4)	0.50621 (15)	0.0904 (5)	0.0085 (6)	
H3	1.2910	0.5020	0.2306	0.010*	
C4	1.2238 (4)	0.45000 (15)	−0.0267 (5)	0.0084 (6)	
C5	1.2108 (4)	0.38080 (15)	0.0705 (5)	0.0092 (6)	
C6	1.1917 (5)	0.45863 (15)	−0.2358 (5)	0.0106 (6)	
H6	1.1553	0.4224	−0.3187	0.013*	
C7	1.2152 (5)	0.52224 (16)	−0.3165 (5)	0.0124 (6)	
H7	1.1968	0.5275	−0.4566	0.015*	

### Atomic displacement parameters (Å^2^)

**Table:**

	*U*^11^	*U*^22^	*U*^33^	*U*^12^	*U*^13^	*U*^23^
Sr1	0.01054 (14)	0.00559 (13)	0.00613 (13)	−0.00023 (12)	0.00104 (9)	0.00023 (12)
N1	0.0119 (13)	0.0086 (13)	0.0083 (12)	−0.0014 (10)	0.0002 (10)	0.0000 (10)
O1	0.0152 (11)	0.0050 (10)	0.0082 (10)	−0.0018 (9)	0.0013 (8)	−0.0012 (9)
O2	0.0198 (12)	0.0098 (11)	0.0086 (11)	−0.0035 (9)	0.0029 (9)	−0.0005 (9)
O3	0.0152 (12)	0.0079 (11)	0.0107 (11)	0.0003 (9)	0.0013 (9)	0.0028 (9)
O4	0.0132 (11)	0.0072 (10)	0.0107 (11)	−0.0008 (8)	0.0013 (9)	−0.0009 (8)
O5	0.0139 (11)	0.0066 (10)	0.0104 (11)	−0.0004 (9)	0.0015 (9)	−0.0028 (8)
O6	0.0136 (12)	0.0105 (11)	0.0277 (14)	0.0013 (9)	0.0050 (10)	0.0045 (10)
C1	0.0102 (15)	0.0073 (14)	0.0089 (14)	0.0007 (12)	0.0013 (11)	−0.0030 (12)
C2	0.0086 (14)	0.0099 (14)	0.0071 (14)	−0.0008 (12)	0.0025 (11)	−0.0002 (12)
C3	0.0107 (15)	0.0082 (14)	0.0065 (14)	0.0010 (12)	0.0010 (11)	0.0018 (11)
C4	0.0083 (14)	0.0044 (13)	0.0128 (15)	−0.0004 (11)	0.0025 (11)	0.0008 (11)
C5	0.0071 (14)	0.0078 (14)	0.0131 (15)	0.0026 (11)	0.0065 (11)	0.0029 (12)
C6	0.0136 (15)	0.0076 (15)	0.0108 (15)	0.0006 (12)	0.0015 (12)	−0.0025 (12)
C7	0.0201 (17)	0.0097 (15)	0.0073 (15)	0.0001 (13)	0.0001 (12)	−0.0003 (12)

### Geometric parameters (Å, °)

**Table:**

Sr1—O6	2.503 (2)	O4—C5	1.260 (4)
Sr1—O2^i^	2.511 (2)	O5—Sr1^ii^	2.688 (2)
Sr1—O1	2.588 (2)	O5—H5B	0.8500
Sr1—O1^ii^	2.600 (2)	O5—H5A	0.8501
Sr1—O5	2.604 (2)	O6—H6B	0.8500
Sr1—O3^iii^	2.636 (2)	O6—H6A	0.8500
Sr1—O5^iv^	2.688 (2)	C1—C2	1.514 (4)
Sr1—N1	2.700 (3)	C2—C3	1.389 (4)
N1—C7	1.341 (4)	C3—C4	1.384 (4)
N1—C2	1.347 (4)	C3—H3	0.9300
O1—C1	1.264 (3)	C4—C6	1.394 (4)
O1—Sr1^iv^	2.600 (2)	C4—C5	1.514 (4)
O2—C1	1.250 (4)	C6—C7	1.377 (4)
O2—Sr1^v^	2.511 (2)	C6—H6	0.9300
O3—C5	1.256 (4)	C7—H7	0.9300
O3—Sr1^iii^	2.636 (2)		
			
O6—Sr1—O2^i^	81.38 (8)	O5^iv^—Sr1—Sr1^ii^	93.74 (4)
O6—Sr1—O1	83.40 (7)	N1—Sr1—Sr1^ii^	144.35 (5)
O2^i^—Sr1—O1	141.34 (7)	Sr1^iv^—Sr1—Sr1^ii^	107.860 (13)
O6—Sr1—O1^ii^	71.92 (7)	C7—N1—C2	117.3 (3)
O2^i^—Sr1—O1^ii^	102.07 (7)	C7—N1—Sr1	123.20 (19)
O1—Sr1—O1^ii^	106.58 (6)	C2—N1—Sr1	116.78 (19)
O6—Sr1—O5	123.32 (7)	C1—O1—Sr1	124.46 (18)
O2^i^—Sr1—O5	71.61 (7)	C1—O1—Sr1^iv^	132.46 (18)
O1—Sr1—O5	144.29 (7)	Sr1—O1—Sr1^iv^	103.01 (7)
O1^ii^—Sr1—O5	66.71 (7)	C1—O2—Sr1^v^	142.38 (19)
O6—Sr1—O3^iii^	156.15 (7)	C5—O3—Sr1^iii^	120.82 (19)
O2^i^—Sr1—O3^iii^	99.20 (7)	Sr1—O5—Sr1^ii^	100.21 (7)
O1—Sr1—O3^iii^	81.53 (7)	Sr1—O5—H5B	122.5
O1^ii^—Sr1—O3^iii^	130.33 (7)	Sr1^ii^—O5—H5B	111.9
O5—Sr1—O3^iii^	78.61 (7)	Sr1—O5—H5A	114.2
O6—Sr1—O5^iv^	119.66 (7)	Sr1^ii^—O5—H5A	84.0
O2^i^—Sr1—O5^iv^	150.91 (7)	H5B—O5—H5A	115.5
O1—Sr1—O5^iv^	65.67 (6)	Sr1—O6—H6B	121.5
O1^ii^—Sr1—O5^iv^	69.75 (6)	Sr1—O6—H6A	120.4
O5—Sr1—O5^iv^	79.68 (5)	H6B—O6—H6A	107.7
O3^iii^—Sr1—O5^iv^	69.94 (7)	O2—C1—O1	126.1 (3)
O6—Sr1—N1	76.37 (8)	O2—C1—C2	116.9 (3)
O2^i^—Sr1—N1	80.71 (7)	O1—C1—C2	117.0 (2)
O1—Sr1—N1	61.18 (7)	N1—C2—C3	122.2 (3)
O1^ii^—Sr1—N1	147.27 (7)	N1—C2—C1	116.4 (3)
O5—Sr1—N1	141.60 (7)	C3—C2—C1	121.4 (3)
O3^iii^—Sr1—N1	80.18 (7)	C4—C3—C2	119.6 (3)
O5^iv^—Sr1—N1	121.72 (7)	C4—C3—H3	120.2
O6—Sr1—Sr1^iv^	84.69 (6)	C2—C3—H3	120.2
O2^i^—Sr1—Sr1^iv^	165.72 (5)	C3—C4—C6	118.4 (3)
O1—Sr1—Sr1^iv^	38.61 (4)	C3—C4—C5	120.5 (3)
O1^ii^—Sr1—Sr1^iv^	70.38 (5)	C6—C4—C5	121.1 (3)
O5—Sr1—Sr1^iv^	114.26 (5)	O3—C5—O4	125.0 (3)
O3^iii^—Sr1—Sr1^iv^	94.81 (5)	O3—C5—C4	117.9 (3)
O5^iv^—Sr1—Sr1^iv^	39.14 (5)	O4—C5—C4	117.0 (3)
N1—Sr1—Sr1^iv^	99.07 (5)	C7—C6—C4	118.3 (3)
O6—Sr1—Sr1^ii^	83.20 (5)	C7—C6—H6	120.9
O2^i^—Sr1—Sr1^ii^	67.43 (5)	C4—C6—H6	120.9
O1—Sr1—Sr1^ii^	144.96 (5)	N1—C7—C6	124.1 (3)
O1^ii^—Sr1—Sr1^ii^	38.38 (5)	N1—C7—H7	117.9
O5—Sr1—Sr1^ii^	40.65 (5)	C6—C7—H7	117.9
O3^iii^—Sr1—Sr1^ii^	119.25 (5)		
			
O6—Sr1—N1—C7	91.7 (2)	O2^i^—Sr1—O5—Sr1^ii^	76.49 (7)
O2^i^—Sr1—N1—C7	8.4 (2)	O1—Sr1—O5—Sr1^ii^	−122.21 (10)
O1—Sr1—N1—C7	−178.4 (3)	O1^ii^—Sr1—O5—Sr1^ii^	−36.03 (6)
O1^ii^—Sr1—N1—C7	106.3 (2)	O3^iii^—Sr1—O5—Sr1^ii^	−179.69 (8)
O5—Sr1—N1—C7	−35.5 (3)	O5^iv^—Sr1—O5—Sr1^ii^	−108.29 (10)
O3^iii^—Sr1—N1—C7	−92.7 (2)	N1—Sr1—O5—Sr1^ii^	122.61 (10)
O5^iv^—Sr1—N1—C7	−151.7 (2)	Sr1^iv^—Sr1—O5—Sr1^ii^	−89.51 (6)
Sr1^iv^—Sr1—N1—C7	173.9 (2)	Sr1^v^—O2—C1—O1	−7.3 (6)
Sr1^ii^—Sr1—N1—C7	34.8 (3)	Sr1^v^—O2—C1—C2	172.1 (2)
O6—Sr1—N1—C2	−107.5 (2)	Sr1—O1—C1—O2	168.8 (2)
O2^i^—Sr1—N1—C2	169.2 (2)	Sr1^iv^—O1—C1—O2	−7.8 (5)
O1—Sr1—N1—C2	−17.58 (19)	Sr1—O1—C1—C2	−10.6 (4)
O1^ii^—Sr1—N1—C2	−92.9 (2)	Sr1^iv^—O1—C1—C2	172.84 (18)
O5—Sr1—N1—C2	125.3 (2)	C7—N1—C2—C3	2.7 (4)
O3^iii^—Sr1—N1—C2	68.1 (2)	Sr1—N1—C2—C3	−159.3 (2)
O5^iv^—Sr1—N1—C2	9.2 (2)	C7—N1—C2—C1	−177.6 (3)
Sr1^iv^—Sr1—N1—C2	−25.3 (2)	Sr1—N1—C2—C1	20.4 (3)
Sr1^ii^—Sr1—N1—C2	−164.33 (16)	O2—C1—C2—N1	172.9 (3)
O6—Sr1—O1—C1	92.9 (2)	O1—C1—C2—N1	−7.7 (4)
O2^i^—Sr1—O1—C1	25.6 (3)	O2—C1—C2—C3	−7.4 (4)
O1^ii^—Sr1—O1—C1	161.74 (19)	O1—C1—C2—C3	172.1 (3)
O5—Sr1—O1—C1	−125.3 (2)	N1—C2—C3—C4	−1.8 (4)
O3^iii^—Sr1—O1—C1	−68.6 (2)	C1—C2—C3—C4	178.5 (3)
O5^iv^—Sr1—O1—C1	−140.3 (2)	C2—C3—C4—C6	−0.8 (4)
N1—Sr1—O1—C1	14.8 (2)	C2—C3—C4—C5	178.4 (3)
Sr1^iv^—Sr1—O1—C1	−177.4 (3)	Sr1^iii^—O3—C5—O4	69.3 (4)
Sr1^ii^—Sr1—O1—C1	161.01 (19)	Sr1^iii^—O3—C5—C4	−109.7 (2)
O6—Sr1—O1—Sr1^iv^	−89.74 (8)	C3—C4—C5—O3	−6.4 (4)
O2^i^—Sr1—O1—Sr1^iv^	−157.03 (9)	C6—C4—C5—O3	172.7 (3)
O1^ii^—Sr1—O1—Sr1^iv^	−20.87 (12)	C3—C4—C5—O4	174.4 (3)
O5—Sr1—O1—Sr1^iv^	52.12 (14)	C6—C4—C5—O4	−6.4 (4)
O3^iii^—Sr1—O1—Sr1^iv^	108.81 (8)	C3—C4—C6—C7	2.4 (4)
O5^iv^—Sr1—O1—Sr1^iv^	37.06 (7)	C5—C4—C6—C7	−176.8 (3)
N1—Sr1—O1—Sr1^iv^	−167.79 (10)	C2—N1—C7—C6	−1.0 (5)
Sr1^ii^—Sr1—O1—Sr1^iv^	−21.60 (12)	Sr1—N1—C7—C6	159.8 (2)
O6—Sr1—O5—Sr1^ii^	10.55 (10)	C4—C6—C7—N1	−1.6 (5)

Symmetry codes: (i) *x*, *y*, *z*−1; (ii) *x*, −*y*+3/2, *z*−1/2; (iii) −*x*+3, −*y*+1, −*z*; (iv) *x*, −*y*+3/2, *z*+1/2; (v) *x*, *y*, *z*+1.

### Hydrogen-bond geometry (Å, °)

**Table:**

*D*—H···*A*	*D*—H	H···*A*	*D*···*A*	*D*—H···*A*
O5—H5B···O4^vi^	0.85	1.95	2.759 (3)	158
O5—H5A···O4^vii^	0.85	1.92	2.730 (3)	160
O5—H5A···O3^vii^	0.85	2.37	3.051 (3)	137
O6—H6B···O3^viii^	0.85	2.12	2.958 (3)	169
O6—H6A···O4^ix^	0.85	2.10	2.833 (3)	144

Symmetry codes: (vi) −*x*+3, −*y*+1, −*z*−1; (vii) −*x*+3, *y*+1/2, −*z*−1/2; (viii) −*x*+2, −*y*+1, −*z*; (ix) −*x*+2, *y*+1/2, −*z*−1/2.
